# Four crystal structures of human LLT1, a ligand of human NKR-P1, in varied glycosylation and oligomerization states

**DOI:** 10.1107/S1399004714027928

**Published:** 2015-02-26

**Authors:** Tereza Skálová, Jan Bláha, Karl Harlos, Jarmila Dušková, Tomáš Koval’, Jan Stránský, Jindřich Hašek, Ondřej Vaněk, Jan Dohnálek

**Affiliations:** a Institute of Biotechnology, Academy of Sciences of the Czech Republic, v.v.i., Vídeňská 1083, 142 20 Praha 4, Czech Republic; b Department of Biochemistry, Faculty of Science, Charles University Prague, Hlavova 8, 128 40 Praha, Czech Republic; c Division of Structural Biology, The Wellcome Trust Centre for Human Genetics, University of Oxford, Roosevelt Drive, Oxford OX3 7BN, England; d Institute of Macromolecular Chemistry, Academy of Sciences of the Czech Republic, v.v.i., Heyrovského nám. 2, 162 06 Praha 6, Czech Republic

**Keywords:** LLT1, C-type lectin-like ligand

## Abstract

Human LLT1 is a C-type lectin-like ligand of NKR-P1 (CD161, gene *KLRB1*), a C-type lectin-like receptor of natural killer cells. Using X-ray diffraction, the first experimental structures of human LLT1 were determined. Four structures of LLT1 under various conditions were determined: monomeric, dimeric deglycosylated after the first *N*-acetylglucosamine unit in two forms and hexameric with homogeneous GlcNAc_2_Man_5_ glycosylation. The dimeric form follows the classical dimerization mode of human CD69. The monomeric form keeps the same fold with the exception of the position of an outer part of the long loop region. The hexamer of glycosylated LLT1 consists of three classical dimers. The hexameric packing may indicate a possible mode of interaction of C-type lectin-like proteins in the glycosylated form.

## Abstract 2

Four crystal structures of human LLT1, a ligand of human NKR-P1, are reported.

## 1. Introduction   

Natural killer (NK) cells are innate immune lymphocytes that possess the ability to recognize and induce the death of a broad range of target cells without prior antigen sensitization, including tumour, virally infected or stressed cells. Apart from direct cell-mediated cytotoxicity, NK cells also participate in the initiation and development of the adaptive immune response through the production and secretion of cytokines (Caligiuri, 2008 ▶; Vivier *et al.*, 2011 ▶). Their function is regulated by a fine balance of signals induced by the interaction of a vast array of surface-activating and surface-inhibitory receptors with ligands on the surface of target cells (Lanier, 2008 ▶; Vivier *et al.*, 2008 ▶; Bartel *et al.*, 2013 ▶). The activation of NK cells and the killing of the target cell is triggered when the expression of cellular health markers (MHC class I-like glycoproteins) that engage the inhibitory NK receptors is too low (‘missing-self’ recognition mode) or when the ligands for activating NK receptors are upregulated, usually in virally infected, stressed or malignant cells (‘induced-self’ mode).

The NK cell receptors are divided into two main structural classes: the immunoglobulin and C-type lectin-like (CTL) superfamilies. The CTL family of NK cells encoded within the natural gene complex (human chromosome 12) comprises proteins related to C-type lectins that have lost the ability to bind carbohydrates but have instead gained the ability to recognize protein ligands (Yokoyama & Plougastel, 2003 ▶; Zelensky & Gready, 2005 ▶; Grobárová *et al.*, 2013 ▶). While certain CTL receptors are known to bind proteins with the MHC class I-like fold (*e.g.* NKG2D, CD94/NKG2A and murine Ly49 receptors; Bartel *et al.*, 2013 ▶), the NKR-P1 receptor subfamily does not share this specificity. Although the ligands for some rodent NKR-P1 receptors are still unknown, it has been shown that many of them recognize genetically and structurally highly related Clr/Ocil CTL receptors from the *CLEC2* gene subfamily (Aust *et al.*, 2009 ▶; Kveberg *et al.*, 2009 ▶). It is now accepted that this unique system of CTL receptor–CTL ligand interaction represents an alternative form of ‘missing-self’ recognition (Bartel *et al.*, 2013 ▶).

Lectin-like transcript 1 (LLT1, gene *CLEC2D*) was identified as a physiological ligand of NKR-P1, the sole described representative of the human NKR-P1 subfamily (CD161, gene *KLRB1*; Aldemir *et al.*, 2005 ▶; Rosen *et al.*, 2005 ▶). LLT1 is expressed primarily on activated lymphocytes (NK cells, T cells and B cells) and antigen-presenting cells (macrophages and dendritic cells; Germain *et al.*, 2011 ▶). Six alternatively spliced transcripts of the *CLEC2D* gene have been identified, with isoform 1 (coding for LLT1) being the only one able to interact with NKR-P1 (Germain *et al.*, 2010 ▶). The engagement of NKR-P1 on the NK cell with LLT1 on the target cell inhibits NK cell cytotoxicity and IFNγ production (Aldemir *et al.*, 2005 ▶; Rosen *et al.*, 2005 ▶, 2008 ▶) and contributes to NK self-tolerance in a similar way to the orthologous rodent NKR-P1B–Clr-b receptor–ligand pair (Voigt *et al.*, 2007 ▶; Fine *et al.*, 2010 ▶; Williams *et al.*, 2012 ▶). It has been shown that human glioblastoma exploits this mechanism by the upregulation of the surface expression of LLT1 to escape the immunological response (Roth *et al.*, 2007 ▶). On the other hand, LLT1 is upregulated in response to both microbial and viral stimuli, and stimulation of NKR-P1-expressing T cells promotes their activation, proliferation and cytokine secretion (Huarte *et al.*, 2008 ▶; Germain *et al.*, 2011 ▶; Satkunanathan *et al.*, 2014 ▶). Thus, LLT1–NKR-P1 signalization may provide a link between pathogen pattern recognition and lymphocyte activation and represents a system that regulates both the innate and the adaptive immune responses.

Recently, we have described the first three-dimensional structure of mouse Clr-g, a representative of the murine CLEC2 family (Skálová *et al.*, 2012 ▶). Similarly to mouse Clr-g, human LLT1 is a type II transmembrane glycoprotein which consists of an N-terminal cytoplasmic chain, transmembrane and stalk regions and a C-terminal CTL domain with two predicted N-linked glycosylation sites. In this study, we present several structures of LLT1 under various conditions: monomeric (LLT1_mono), dimeric deglycosylated after the first *N*-acetyl­glucosamine unit (LLT1_D1 and LLT1_D2) and dimeric (packed into hexamers) with homogeneous GlcNAc_2_Man_5_ glycosylation (LLT1_glyco). All structures originate from protein expressed in HEK293S GnTI^−^ cells (Reeves *et al.*, 2002 ▶). The dimeric form follows the classical dimerization mode of human CD69 (Natarajan *et al.*, 2000 ▶; Llera *et al.*, 2001 ▶; Vaněk *et al.*, 2008 ▶; Kolenko *et al.*, 2009 ▶).

## 2. Materials and methods   

### 2.1. Protein expression and purification   

The lectin-like domain of LLT1 was produced in HEK293S GnTI^−^ cells as described by Bláha *et al.* (2015 ▶). Briefly, the expression construct corresponding to the CTL extracellular domain of LLT1 (Gln72–Val191) with a His176 to Cys176 mutation was cloned into the pTT28 plasmid, a derivative of the pTT5 plasmid (Durocher *et al.*, 2002 ▶) with an altered cloning site (Bláha *et al.*, 2015 ▶). A suspension culture of HEK293S GnTI^−^ cells (Reeves *et al.*, 2002 ▶) was transiently transfected with a 1:3 mixture of the expression plasmid and 25 kDa linear polyethyleneimine. The resulting protein contained an N-terminal secretion leader and a C-terminal His_8_ tag. 5–7 d post-transfection, the secreted recombinant protein was purified from the harvested production medium by two-step chromatography using batch IMAC on Talon resin (Clontech) followed by size-exclusion chromatography on a Superdex 200 10/300 GL column (GE Healthcare) in 10 m*M* HEPES pH 7.5 with 150 m*M* NaCl and 10 m*M* NaN_3_. The final product was concentrated to 20 mg ml^−1^ using an Amicon Ultra concentrator (10 kDa molecular-weight cutoff; Merck Millipore).

For deglycosylation, GST-fused Endo F1 (Grueninger-Leitch *et al.*, 1996 ▶) was added to the concentrated protein solution in a 1:200 weight ratio and incubated overnight at room temperature. The slight precipitate that developed after deglycosylation was dissolved by the addition of l-arginine–HCl to a concentration of 0.4 *M* before setting up the crystallization drops.

The C-type lectin-like ectodomain of LLT1 contains two of the three canonical disulfide bridges (Cys75–Cys86 and Cys103–Cys184) found in homologous CTL receptors. Multiple alignment analysis showed that the formation of the third canonical disulfide bridge is impaired by the absence of the sixth evolutionarily conserved Cys residue, which is substituted by His176 in the wild-type LLT1 sequence. Based on previous results (Kamishikiryo *et al.*, 2011 ▶), we decided to clone and express the extracellular part (Gln72–Val191) of LLT1 with the His176 to Cys176 mutation. This mutation dramatically improved the stability, homogeneity and yield of the product (Bláha *et al.*, 2015 ▶). Expression in HEK293S GnTI^−^ cells and subsequent purification provided highly pure protein with artificially homogeneous (GlcNAc_2_Man_5_) N-linked glycosylation with a typical yield of 3 mg of purified protein per litre of production culture. The disulfide-bond pattern as well as the occupancy of both N-glycosylation sites was verified by mass spectrometry (Bláha *et al.*, 2015 ▶).

### 2.2. Crystallization and data collection   

#### 2.2.1. LLT1_mono   

The protein (diluted to 14 mg ml^−1^ in 0.4 *M* arginine, 4 m*M* HEPES, 120 m*M* NaCl, 4 m*M* NaN_3_ pH 7.5) was crystallized using the sitting-drop vapour-diffusion method. Drops (100 nl reservoir solution and 100 nl protein solution) were set up using a Cartesian Honeybee 961 robot (Genomic Solutions) at 294 K. The reservoir consisted of 2 *M* ammonium sulfate, 0.1 *M* sodium citrate pH 3.5. A crystal with the shape of a hexagonal plate (dimensions of 100 × 100 × 10 µm) was cryoprotected by soaking in the reservoir solution with the addition of 25% glycerol. The diffraction data were measured on a split segment of the multi-crystal on beamline I02 of Diamond Light Source (DLS) using an ADSC Q315 CCD detector at 100 K.

#### 2.2.2. LLT1_D1 and LLT1_D2   

The protein was crystallized as above. The reservoir consisted of 30%(*w*/*v*) polyethylene glycol (PEG) 6000, 0.1 *M* HEPES pH 7.0. A cuboid-shaped crystal of dimensions ∼60 × 60 × 120 µm was cryoprotected as above. The data were measured on beamline I04-1 at DLS using a PILATUS 2M detector at 100 K. Both data sets were collected using crystals from the same crystallization condition. Interestingly, both crystals belonged to the same space group, *P*2_1_2_1_2_1_, but the crystals differed in solvent content (36% for LLT1_D1 and 25% for LLT1_D2) and in unit-cell parameters (a difference of 4 Å in *b* and 8 Å in *c*).

#### 2.2.3. LLT1_glyco   

Protein diluted to 14 mg ml^−1^ in 10 m*M* HEPES, 150 m*M* NaCl, 10 m*M* NaN_3_ pH 7.5 was crystallized using the hanging-drop vapour-diffusion method (with drops consisting of 1 µl reservoir solution and 1 µl protein solution) with reservoir consisting of 40%(*v*/*v*) PEG 300, 0.1 *M* citrate–phosphate buffer pH 4.2 at a temperature of 288 K. A rod-shaped crystal of dimensions ∼200 × 50 × 50 µm was vitrified without cryoprotection. Diffraction data were measured on BM 14.1 of the BESSY II synchrotron-radiation source (Mueller *et al.*, 2012 ▶) at the Hemholtz-Zentrum Berlin using a MAR Mosaic 225 CCD detector at 100 K.

### 2.3. Structure determination   

The data sets for LLT1_mono and LLT1_glyco were processed using the *HKL*-2000 package (*DENZO* and *SCALEPACK*; Otwinowski & Minor, 1997 ▶). Intensities were converted to amplitudes and imported into the CCP4 format with *TRUNCATE* (French & Wilson, 1978 ▶). The data for LLT1_D1 and LLT1_D2 were processed in *MOSFLM* (Leslie & Powell, 2007 ▶) using the *iMosflm* interface (Battye *et al.*, 2011 ▶) and were scaled in *AIMLESS* (Evans & Murshudov, 2013 ▶). The data parameters are shown in Table 1 ▶.

The data for LLT1_glyco showed strong anisotropy; therefore, the data processed in *DENZO* and *SCALEPACK* were then rescaled using the *Diffraction Anisotropy Server* (http://services.mbi.ucla.edu/anisoscale/; Strong *et al.*, 2006 ▶). The processed data comprised 4186 reflections. An isotropic *B* factor of −23.39 Å^2^ was applied to restore the magnitude of the high-resolution reflections diminished by anisotropic scaling.

The phase problem for the LLT1_D1, LLT1_D2 and LLT1_glyco structures was solved by molecular replacement in *BALBES* (Long *et al.*, 2008 ▶; Keegan *et al.*, 2013 ▶) using the structure of human CD69 as a search model (Kolenko *et al.*, 2009 ▶; PDB entry 3hup). The structure of LLT1_mono was solved in *MOLREP* (Vagin & Teplyakov, 2010 ▶) using one chain of LLT1_glyco as a search model.

All of the structures were refined in *REFMAC*5 (Murshudov *et al.*, 2011 ▶) with manual changes performed in *Coot* (Emsley *et al.*, 2010 ▶). Structure parameters are shown in Table 1 ▶. After several cycles of rigid-body refinement, the structures were refined using restrained refinement including H atoms (not deposited in the Protein Data Bank). Non­crystallographic symmetry between two monomers of the dimer was applied in the cases of LLT1_D1, LLT1_D2 and LLT1_glyco in the first steps of the refinement. The refinement was performed with the omission of 5% of the reflections (test set with *R*_free_ flag). In the case of LLT1_glyco with a lower resolution and a lower number of reflections, 6% of the reflections (223 reflections) were used for the test set. The last cycle of refinement of all four structures was performed with all reflections including the test set.

The choice of the correct space group for LLT1_glyco was ambiguous and structure refinement was tested in 16 space groups alternative to *P*2_1_ using *Zanuda* (Lebedev & Isupov, 2014 ▶) and then also manually in *P*2_1_, *C*222, *C*222_1_, *P*6_3_ and *P*6_3_22. Finally, *P*6_3_22 was chosen as the correct space group because lower symmetry space groups did not show apparent differences among the molecules in the asymmetric unit and refinement was less stable.

### 2.4. Structure quality: geometry and electron density   

#### 2.4.1. LLT1_mono   

According to the *MolProbity* Ramachandran plot (Chen *et al.*, 2010 ▶), 95% of the residues of the structure lie in the favoured regions. There is one outlier, Gln83. This is a residue in a loop with an alternative conformation of the main chain. The residue is in good agreement with the 2*mF*_o_ − *DF*_c_ and *mF*_o_ − *DF*_c_ electron-density Fourier maps.

One sulfate anion is localized in the structure, bound to Lys181 N^ζ^, Ser129 N and Ser129 O^γ^. The peptide bond Lys126-Gly127 in the vicinity of the sulfate anion has *cis*–*trans* alternative conformations.

#### 2.4.2. LLT1_D1   

This structure with high resolution has residues generally well localized in 2*mF*_o_ − *DF*_c_ and *mF*_o_ − *DF*_c_ maps; however, the Fourier maps have lower quality in the region of the long loop (residues 137–160), mainly in chain *A*. According to the *MolProbity* Ramachandran plot, 98% of the residues lie in the favoured regions and there is only one outlier, residue *A*Gln139, which is in a turn of a loop.

There are unmodelled peaks in the 2*mF*_o_ − *DF*_c_ Fourier maps. The electron density for the side chain of Lys186 in both chains is elongated, which may indicate covalent modification of this lysine. Moreover, arginine as added to the LLT1 sample after deglycosylation to improve protein folding is probably localized on the protein surface, bound by hydrogen bonds to residues *B*Asp104 and *B*Asp109, but the electron density is not sufficiently clear to build the whole arginine residue.

Surprisingly, the conformation of the Gln83-Arg84 peptide bond differs significantly between chain *A* (Gln83 torsion angles ϕ = 47°, ψ = 51°) and chain *B* (ϕ = 54°, ψ = −122°). Electron density is well defined in both cases. In chain *B* the value lies in the allowed region of the Ramachandran plot and *B*Gln83 O forms a water-mediated hydrogen bond to *A*Cys75 N. In chain *A*, the torsion-angle values lie in the favoured region of the Ramachandran plot but the connection formed between chains *A* and *B* is weaker: it is mediated by hydrogen bonds formed by two water molecules connecting *A*Arg84 N and *B*Cys75 N.

#### 2.4.3. LLT1_D2   

Of the four presented structures, this structure has the best quality electron density because of its high resolution (1.8 Å) and low solvent content (25%). The long loop region of both chains is in contact with symmetry-related molecules in the crystal and is better localized in electron density than in the other presented structures. According to the *MolProbity* Ramachandran plot, 98% of residues lie in the favoured regions and there are no outliers.

Unlike in LLT1_D1, there are no signs of Lys186 modification. Solvent arginine bound to *B*Asp104 and *B*Asp109 could be present, but is less apparent than in LLT1_D1. The Gln83-Arg84 peptide bond is modelled in the same orientation in both chains, even though there are some signs of the alternative conformation in chain *A*.

#### 2.4.4. LLT1_glyco   

The structure of glycosylated LLT1 has lower resolution (2.7 Å) and the data have an anisotropic character. According to the *MolProbity* Ramachandran plot, 87% of residues lie in the favoured regions and there are two outliers, *A*Ser105 and *A*Ile146, both of which are in turns of different loops.

Tyr88 has elongated electron density which may indicate covalent modification of this tyrosine. Some positive signal in the *mF*_o_ − *DF*_c_ map behind the hydroxyl group of Tyr88 is also present in chain *B* of LLT1_D1.

### 2.5. Electrostatic potential   

The electrostatic potential of LLT1_D2 was computed by solving the Poisson–Boltzmann equation in *APBS* (Baker *et al.*, 2001 ▶). H atoms were added in *PROPKA* (Li *et al.*, 2005 ▶), including optimization of hydrogen bonds for the protein at pH 7.5. Partial charges were assigned in *PDB*2*PQR* (Dolinsky *et al.*, 2007 ▶) based on the AMBER potential. The Poisson–Boltzmann equation was solved using values of the dielectric constants of ∊(solvent) = 78.54 and ∊(protein) = 2 and assuming a 0.225 *M* concentration of ions in solution with charges +*e* and −*e* and radius 2 Å.

### 2.6. Sedimentation analysis   

The oligomeric state of the produced protein was analyzed in a ProteomeLab XL-I analytical ultracentrifuge equipped with an An-50 Ti rotor (Beckman Coulter, USA) using a sedimentation-velocity experiment. Samples of glycosylated LLT1 diluted with the buffer used in size-exclusion chromatography to the desired concentration were spun at 50 000 rev min^−1^ at 20°C and 100 scans with 0.003 cm spatial resolution were recorded using absorbance optics at 280 nm. Buffer density and protein partial specific volume were estimated in *SEDNTERP* (http://sednterp.unh.edu/). The data were analyzed using *SEDPHAT* (Schuck, 2003 ▶) using the continuous size-distribution model and the monomer–dimer self-association model.

### 2.7. Dynamic light scattering   

Measurement of dynamic light scattering was performed with a Zetasizer Nano (Malvern Instruments) and a 45 µl quartz cuvette with ∼40 µl LLT1 solution (sample corresponding to LLT1_glyco) diluted to 1 mg ml^−1^ in a buffer solution consisting of 10 m*M* HEPES, 150 m*M* NaCl, 10 m*M* NaN_3_ pH 7.5.

### 2.8. Figure preparation   

**Figure 1:**
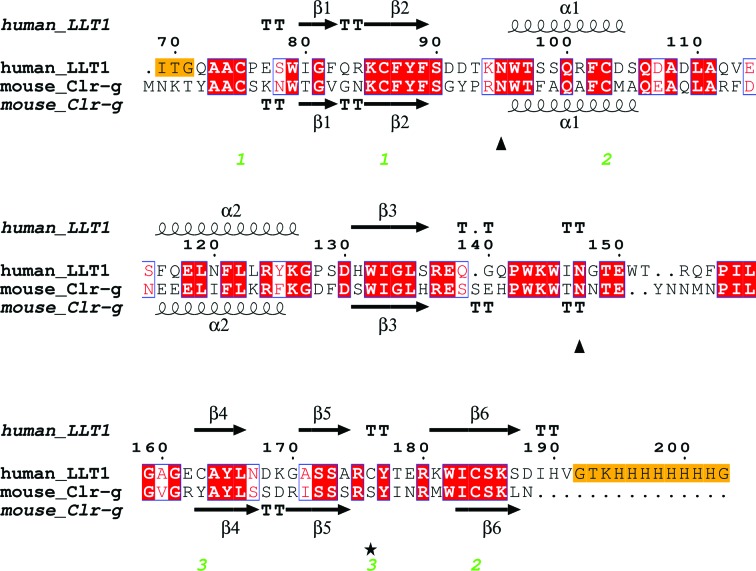
The LLT1 expression construct used for crystallization and its sequence alignment (*Clustal Omega*; Thompson *et al.*, 1994 ▶; Sievers *et al.*, 2011 ▶) with mouse Clr-g. The H176C mutation is denoted by an asterisk, glycosylated Asn residues are denoted by triangles and the three pairs of disulfide bonds in LLT1 are numbered. Uncleaved residues of the N-terminal signal sequence and the C-terminal tag are in orange boxes. Secondary structure was assigned by *DSSP* (Kabsch & Sander, 1983 ▶).

**Figure 2:**
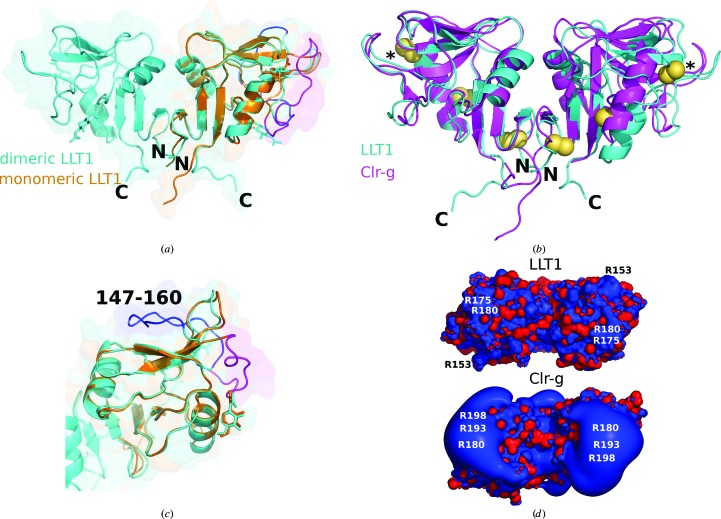
(*a*) Structures of the dimeric (LLT1_D2, cyan) and monomeric (LLT1_mono, orange) forms of LLT1. The loop is in its standard dimeric position in LLT1_D2 (blue) and is in the swapped position in LLT1_mono (magenta). The N- and C-termini of LLT1_D2 are denoted. (*b*) Comparison of the structure of LLT1_D2 (cyan) with its mouse orthologue Clr-g (PDB entry 3rs1, magenta). The superposition was performed in *Coot* by SSM of chains *A* (on the left). The N- and C-termini of LLT1_D2 are denoted. Disulfide bonds are shown as yellow spheres, with the artificial disulfide bonds induced by the H176C mutation denoted by asterisks. (*c*) A detailed view of the loop position discussed in (*a*). (*d*) Electrostatic equipotential surfaces of LLT1_D2 (top) and mouse Clr-g (bottom). They are displayed at the 3 *kT*/e (blue) and −3*kT*/e (red) levels as computed in *APBS* (Baker *et al.*, 2001 ▶). The view is from the ‘top’ of the dimer, *i.e.* from the termini-distal side.

**Figure 3:**
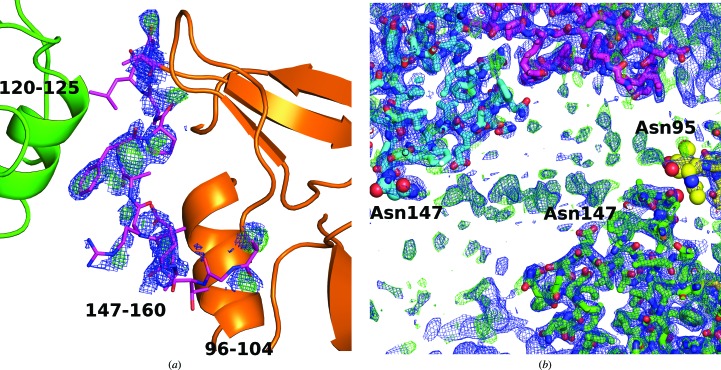
(*a*) Electron-density OMIT maps for the flexible loop (residues 147–160) placed in an unusual position in the monomeric structure of LLT1 (LLT1_mono). 2*mF*_o_ − *DF*_c_ (blue, 0.8σ) and *mF*_o_ − *DF*_c_ (green, 2.5 σ) Fourier maps are drawn up to a distance of 1.6 Å from the atoms of residues 147–160. The residues are better localized in the middle of the loop, where they are stabilized by the nearby α-helix of a neighbouring molecule in the crystal (green). The loop is not modelled in the deposited structure because weak signal in this region causes unstable refinement. (*b*) Two neighbouring hexamers in the crystal of LLT1_glyco are connected by weak electron density belonging to the glycan chains. The glycosylated residues and GlcNAc units are shown as spheres. 2*mF*_o_ − *DF*_c_ (blue, 0.7σ) and *mF*_o_ − *DF*_c_ (green, 1.8σ) Fourier maps are shown.

**Figure 4:**
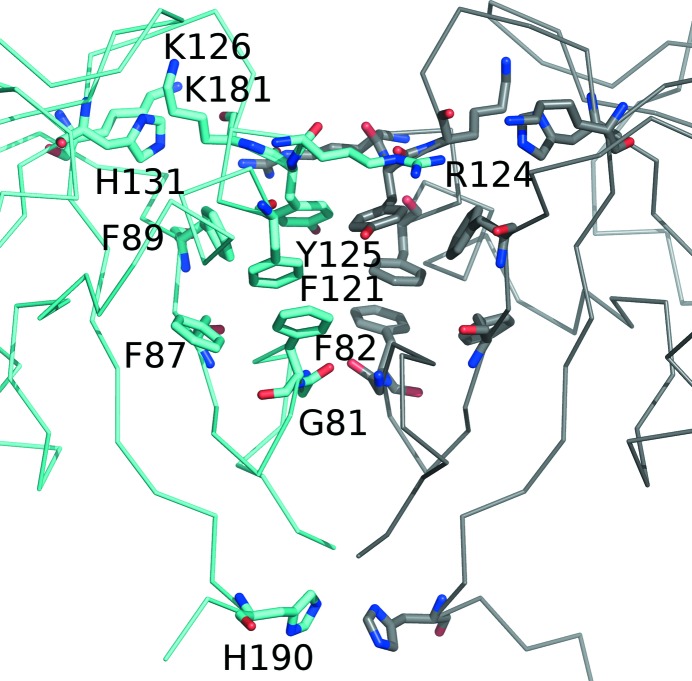
A detailed view of the LLT1 dimer interface (structure LLT1_D2).

**Figure 5:**
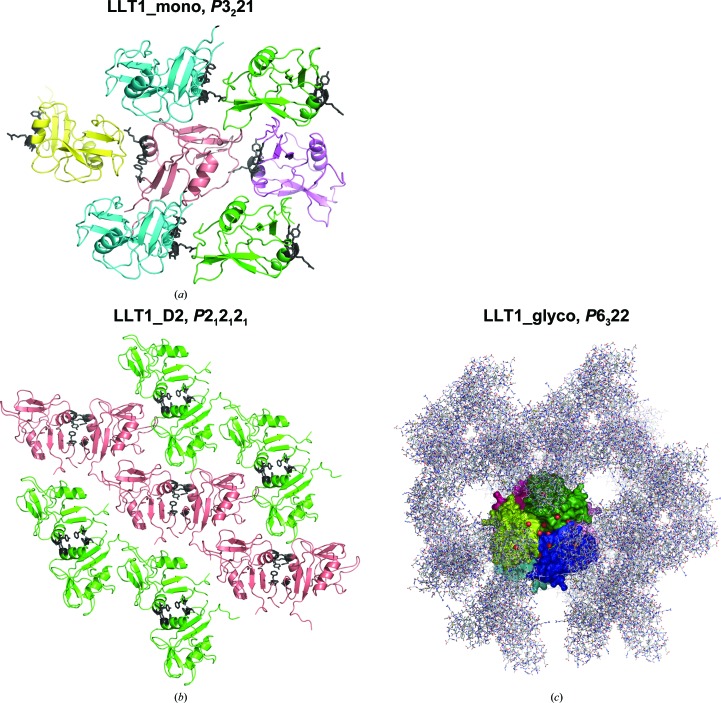
Crystal packing of the presented LLT1 structures. (*a*) LLT1_mono. Colours are according to the orientation of the protein molecule in the crystal. Residues that would form the dimer interface in the case of a dimer (Gly81, Phe82, Phe121, Arg124 and Tyr125) are shown as black sticks. (*b*) LLT1_D2 with the same colour coding. The dimer interface is shown as black sticks. LLT1_D1 has similar packing as LLT1_D2. (*c*) LLT1_glyco. One hexamer, containing three dimers of LLT1, is represented by a molecular surface colour-coded by individual protein chains and viewed along the crystallographic threefold axis. Surrounding symmetry-related chains are represented as sticks.

**Figure 6:**
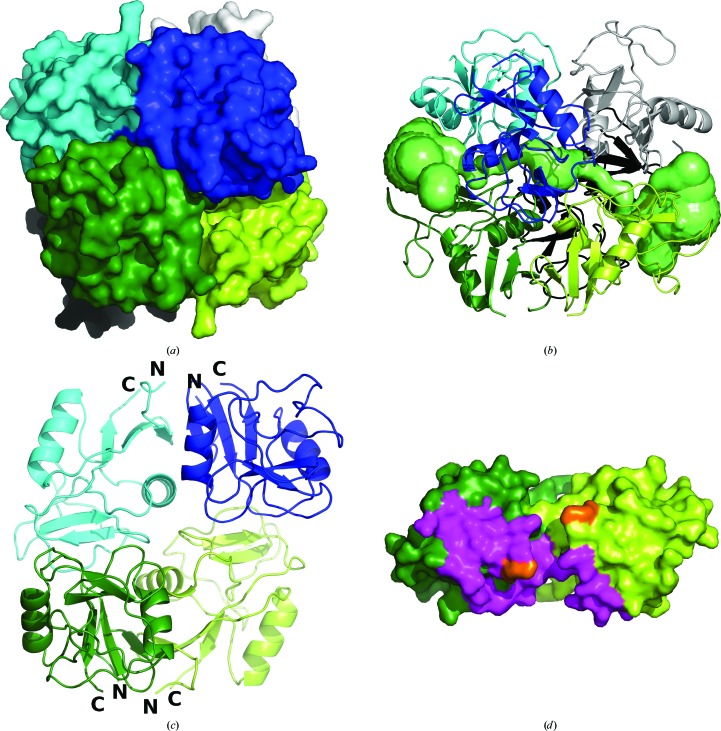
The fully glycosylated form of LLT1 forms hexamers in crystals which are an assembly of three classical dimers. The dimers are distinguished by colour (dark and light green, dark and light blue and black and white). (*a*) A general view of the hexamer. (*b*) Visualization of the tunnel and the central cavity of the hexamer (computed in *CAVER*). A view perpendicular to the threefold axis, which is the direction of the tunnel. (*c*) Dimer–dimer contacts in the hexamer. One dimer of the hexamer is omitted to show the mutual orientation of two dimers. The N- and C-termini of the chains are denoted. (*d*) Contact residues of a dimer with its neighbour in the hexamer. Residues forming the LLT1 dimer–dimer contact up to a distance of 5 Å are shown in magenta. Residue Lys169 (important for interaction in the NKR-P1–LLT1 complex) is coloured orange. The contact residues are localized on the termini-distal side. Interacting residues were identified using *NCONTACT* from the *CCP*4 package (Winn *et al.*, 2011 ▶).

Fig. 1 was prepared using *ESPript* (http://espript.ibcp.fr; Gouet *et al.*, 2003 ▶). Figs. 2–6 and Supplementary Fig. S1 were prepared in *PyMOL* (Schrödinger; http://www.pymol.org).

## 3. Results and discussion   

The extracellular part of LLT1 was expressed in the HEK293S GnTI^−^ cell line and was purified. Three crystal forms were grown at pH 3.5, 4.2 and 7.0 and led to four structures of LLT1: monomeric, dimeric (with altered crystal packing) and dimeric packed as hexamers. The hexameric structure of LLT1 has GlcNAc_2_Man_5_ N-glycosylation; in the other three LLT1 structures the protein is deglycosylated beyond the first GlcNAc.

### 3.1. Overview of the crystal structures of LLT1   

The crystallized extracellular part of human LLT1 corresponds to the sequence deposited in the UniProt database under code Q9UHP7, isoform 1, starting with Gln72 and with a His176 to Cys176 mutation (for the formation of a Cys163–Cys176 disulfide bond for greater stabilization of the protein fold) and with the addition of ITG (the remnant of a secretion leader) at the beginning of the chain and of a GTKHHHHHH­HHG tag at the end of the chain (Fig. 1 ▶). The calculated molecular weight of this construct is 15.7 kDa (18.1 kDa taking N-glycosyl­ation into account).

The extracellular part of human LLT1 has the C-type lectin-like fold (Fig. 2 ▶ *a*) with two α-helices, two antiparallel β-sheets and three disulfide bonds (Cys70–Cys86, Cys103–Cys184 and Cys163–Cys176). The third (artificial) disulfide bond induced by the mutation of His176 to Cys176 is located in the same position as the third disulfide bond occurring naturally in CD69. The overall manner of dimerization of both its glycosylated and deglycosylated dimeric forms (produced in a human cell line in a close-to-native form) corresponds to the classical dimerization mode of human CD69 or of mouse Clr-g (one of the mouse orthologues of LLT1) both produced in bacteria and refolded from inclusion bodies. The N- and C-termini of both chains in this type of dimer are localized on one side (Fig. 2 ▶ *b*) and their position allows protein anchoring in the cell membrane, which supports the biological relevancy of this dimer.

The protein used to obtain the LLT1_glyco structure contained homogeneous GlcNAc_2_Man_5_ glycosylation, while the protein used to obtain the LLT1_mono, LLT1_D1 and LLT1_D2 structures was deglycosylated beyond the first GlcNAc. However, only the first GlcNAc unit could be modelled in LLT1_glyco. The subsequent oligosaccharide moieties cannot be modelled but are visible at low electron-density levels.

In LLT1_mono, residues 147–160 (the outer loop of the long loop region) are poorly visible and were not built in the deposited structure. However, the approximate position of the loop is apparent in the electron density and differs significantly from its position in LLT1 in its dimeric form (Figs. 2 ▶ *c* and 3 ▶ *a*). The structure of LLT1_mono with the approximate position of the loop is available from the authors upon request.

### 3.2. Comparison of monomeric and dimeric LLT1   

Superposition of chain *A* of LLT1_D2 on LLT1_mono by secondary-structure matching in *Coot* gives an r.m.s. deviation of C^α^atoms of 0.6 Å. The core of the monomer and the dimer has the same structure, but there are some structural differences in the outer parts of the CTL domain.

As mentioned above, there is an important difference in the placement of the outer loop (residues 147–160) of the long loop region (residues 137–160). While in LLT1_D2 this loop lies in the direction of β-sheet 171–174 and α-helix 116–126 of the same chain, as is common in the structures of similar proteins (*e.g.* the CD69 dimer), in LLT1_mono the loop is turned to the opposite side with regard to the inner loop 135–145: it is situated near to α-helix 96–104 of the same chain and makes contact with α-helix 120–125 of a neighbouring monomer in the crystal (Figs. 2 ▶ *c* and 3 ▶ *a*).

There are also other structural differences between the monomer and dimer structures: the C-terminal part of the main chain, residues 190–194, is placed differently (Fig. 2 ▶ *a*) and residues around Gln139 are mutually shifted; the distance between the C^α^ atoms in the monomeric and dimeric forms is 1.9 Å.

### 3.3. Dimer interface in dimeric LLT1   

LLT1_glyco, LLT1_D1 and LLT1_D2 share essentially the same structure at the level of the dimer; however, they differ slightly in the mutual orientation of the monomers within a dimer. This difference is especially apparent when the dimers are overlapped by only one chain: the rotation necessary to superimpose chain *A* onto chain *B* of the selected protein is exactly 180° for the LLT1_glyco dimer crystallized in space group *P*6_3_22, in which the chains are related by a crystallo­graphic rotation axis. In contrast, it is 173.7° for LLT1_D1 and 177.7° for LLT1_D2, both of which crystallized in space group *P*2_1_2_1_2_1_ with one dimer in the asymmetric unit. Owing to these differences in mutual orientation, there are differences in the area of the dimer interface (obtained using the *PISA* server; Krissinel & Henrick, 2007 ▶): the size of the contact area is 530 Å^2^ for LLT1_glyco (8% of the monomer surface area), 740 Å^2^ for LLT1_D2 (10% of the monomer surface area) and 810 Å^2^ for LLT1_D1 (11% of the monomer surface area).

The dimeric interface in all three LLT1 dimers is based on a hydrophobic core formed by phenyl rings, analogous to that observed in similar CTL dimers: Phe121, Tyr125, Phe89, Phe87 and Phe82 (Fig. 4 ▶). Additionally, there is an interaction formed by π-stacking of arginine residue Arg124 of both chains, and a pair of His190 residues forms a partial stacking interaction in the termini region (the part of the CTL domain oriented towards the cell membrane).

There are six common hydrogen bonds that connect the monomers in all three dimers: *A*Gly81 N–*B*Gly81 O, *A*Arg124 N^∊^–*B*Tyr125 O, *A*Arg124 N^η1^–*B*Lys126 O and the three analogous hydrogen bonds with exchanged chains. Additionally, there are some hydrogen bonds specific to individual cases: one additional hydrogen bond in the dimeric interface of LLT1_glyco, two hydrogen bonds in LLT1_D2 and six in LLT1_D1.

This variability of the mutual chain orientation has been discussed previously for similar CTL protein dimers (CD94/NKG2A, Clr-g and CD69; Sullivan *et al.*, 2007 ▶; Skálová *et al.*, 2012 ▶). Considering the flexibility of the dimer interface, it seems that the flexible hydrophobic interactions are the most important contact for the existence of the dimer, its stability and its ability to bind a protein partner. These CTL dimers differ in their main purpose, which is surface partner binding, from for example the small aspartic proteases (such as HIV-1 protease), which in contrast require a strictly defined binding site at the dimer interface providing an environment for a specific peptide-bond cleavage. Increased flexibility within the dimer interface would be destructive for the function of proteases, unlike in this case of CTL protein–CTL ligand complexation, where the flexibility and ability to adapt the dimer shape to enable protein partner binding could be an advantage.

### 3.4. Crystal packing of monomeric LLT1   

The crystal packing of LLT1_mono was investigated to confirm that the form of the protein in this structure is really monomeric and that no possible partner participating in oligomer formation can be found among the symmetry-related copies. The crystal packing is shown in Fig. 5 ▶(*a*). The classical dimerization interface is left open to the solvent, without any crystal contacts. The largest intermolecular interface in the crystal (according to the *PISA* server; Krissinel & Henrick, 2007 ▶) has a surface area of 724 Å^2^ (11% of the monomer surface area) and includes ten hydrogen bonds. The following interface is much weaker (372 Å^2^). The largest intermolecular contact is formed by the C- and N-termini of the chain with the region above the β-sheet of the neighbouring molecule where the outer loop of the long loop region is usually localized. In this structure, the outer loop (147–160) of the long loop region is turned over towards the other side along the inner loop and the terminal region of a neighbouring molecule takes its place (Figs. 2 ▶ *c* and 5 ▶ *a*). This contact with the C- and N-termini cannot be a biologically relevant contact, because in the cell the N-terminal chain continues to the membrane. This leads to the conclusion that in this structure the LLT1 monomer does not form any biologically relevant contacts.

### 3.5. Crystal packing of dimeric LLT1   

The strongest dimer–dimer contact in the LLT1_D1 and LLT1_D2 crystals is the contact of the turns of three loops (around residues 139, 160 and 177) with a ‘binding pocket’ at the dimer interface (in the part distant from the N- and C-termini; Fig. 5 ▶ *b*). The contact has an area of 620 Å^2^ and includes 15 hydrogen bonds. An *N*-acetylglucosamine unit bound to Asn95 is also in this region and is close to the ‘binding pocket’. This type of crystal packing would be much more complicated or impossible in the case where the protein was not deglycosylated after the first GlcNAc unit.

In our structural study of the Clr-g receptor (Skálová *et al.*, 2012 ▶), the strongest contact in the crystal was also connected with a bond to this ‘binding pocket’ at the dimer interface. In that case, it was the truncated N-terminus of the Clr-g chain making a tight interaction with the neighbouring dimer interface (the ‘binding pocket’), a clearly strong but biologically irrelevant interaction.

### 3.6. Role of pH in the formation of dimers   

The monomeric form of LLT1 was crystallized at pH 3.5 and the changed protonation state at low pH corresponds with the changed preferred intermolecular contacts and the formation of oligomers.

As an example, residue His190, localized near the C-terminus in the dimerization interface, is charged at low pH and destabilizes this part of the interface. It is apparent from the structures that His190 is neutral in LLT1_D1 and LLT1_D2 (pH 7.0) and is close to His190 (3.1 and 3.3 Å) from the opposite chain, participating in a partial stacking inter­action. In LLT1_glyco (pH 4.2) His190 is not localized in electron density. In LLT1_mono His190 is very likely to be protonated as it forms a hydrogen bond to the N-terminal Gln72 O of the same chain.

Residues His131 and Lys181 are found in LLT1_D1 and LLT1_D2 close (with regard to interaction of charges, ∼5 Å) to the site in which the guanidinium group of Arg124 reaches over from the opposite chain to ‘lock’ the dimer interface, which is otherwise formed mainly by the hydrophobic core. Arg124 stacks on the side chain of Tyr125 and forms additional hydrogen bonds. In the low-pH structure LLT1_mono, His131 is charged and this site attracts an SO_4_^2−^ ion. Under these conditions the positive charge of Lys181 and His131 repels Arg124 from its standard position and thus contributes to dimer destabilization.

As LLT1 is expected to function as a dimer, very low pH values would disable its dimerization and its function as such. A tumour environment often shows decreased pH, but not such drastically lowered values, and a decrease of pH in some cases also leads to higher activity of cytotoxic cells. Given that we observe standard dimers at pH 4.2 in the LLT1_glyco structure and only the extreme pH of 3.5 in LLT1_mono leads to dimer disruption, this direct observation of behaviour at extremely low pH is not relevant to normal biological limits.

### 3.7. Hexamers of LLT1 with GlcNAc_2_Man_5_ glycosylation   

The LLT1_glyco structure is packed into hexamers (Figs. 5 ▶ *c* and 6 ▶). The hexamer is formed by three dimers, which are similar to those of LLT1_D1 and LLT1_D2. The hexamer has one threefold and three perpendicular twofold axes of symmetry (point symmetry 32, where the asymmetric unit is one monomer). The hexamer of ∼70 Å diameter contains a central cavity of about 10 Å in diameter. The cavity is formed by the surface depressions of the three participating dimers found on the termini-distal surface. Six Pro128 residues are exposed into the cavity and six Asp168 and Lys169 residues with their side chains near to the proline residues together form the border of the cavity. The cavity is connected to the exterior of the hexamer by a channel along the threefold axis of the hexamer (Fig. 6 ▶ *b*; the channel shape was computed in *CAVER*; Chovancova *et al.*, 2012 ▶).

The tightest monomer–monomer contact in the hexamer is the standard dimerization contact (530 Å^2^). The second largest contact has practically the same area (528 Å^2^) but lacks hydrogen bonds. The third largest contact, with a surface area of 377 Å^2^, involves six hydrogen bonds (*A*Cys176 N–*B*Glu138 O^∊1^, *A*Tyr177 N–*B*Glu138 O^∊1^, *A*Arg101 N^η2^–*B*Asn147 O and three analogous hydrogen bonds with exchanged chains). The second and the third strongest contacts are formed to molecules of the neighbouring dimer so that the dimer–dimer contact in the hexamer is relatively strong and these additional interactions explain the weakened interactions of monomers in the LLT1_glyco dimerization interface, as described in §[Sec sec3.3]3.3.

The N- and C-termini of the extracellular part of LLT1 are localized on the surface of the hexamer, but in different directions for different dimers (Fig. 6 ▶ *c*). This indicates that LLT1 present on the surface of one cell cannot form such hexamers. However, the dimer packing into the hexamers may indicate a possible method of NK receptor–ligand interaction between two cells (see §[Sec sec3.11]3.11).

### 3.8. Role of glycosylation in LLT1_glyco   

In the hexamer, the glycosylated Asn residues Asn95 and Asn147 are localized on its surface. Three Asn95 residues related by the threefold axis are at a mutual distance of 18 Å and are placed relatively close to the central channel. Three Asn147 residues located ∼18 Å from the Asn95 residues in the direction away from the central channel are distant from each other. The other three Asn95 and three Asn147 residues are in the same formation on the opposite side of the hexamer.

However, Asn147 is very close to Asn95 from a symmetry-related hexamer in the unit cell: the distance between Asn147 O and Asn95 O^δ1^ from a symmetry-related molecule is 7.7 Å (Fig. 3 ▶ *b*). In spite of the fact that it is not possible to model any unit of glycosylation well at Asn147 because the electron density is not sufficiently unambiguous, there is apparent continuous electron density between Asn147 and Asn147 from the neighbouring hexamer (the distance between the Asn147 N^δ2^ atoms is 33 Å; Fig. 3 ▶ *b*) with peaks of up to 1.3σ in 2*mF*_o_ − *DF*_c_ and 3.6σ in *mF*_o_ − *DF*_c_ maps, indicating that the oligosaccharide chains interconnect molecules and indeed the individual hexamers in the crystal. Thus, we conclude that we do not observe any role of glycans in the formation of the hexamer; however, it is evident that the glycans contribute to the packing of the hexamers into the crystal lattice.

### 3.9. Influence of the His176Cys mutation on the LLT1 structure   

The mutated residue 176 is localized on the surface of LLT1 near the long loop region. It is distant from the N- and C-terminal parts and also does not lie in the dimerization interface. The additional S—S bond involving Cys176 stabilizes the protein fold. It is probable that the His176 form of LLT1 would have a longer flexible part of the loop than the flexible part 147–160 observed in the structure of the Cys176 variant. The residue lies in a crystal contact region in the dimeric and hexameric structures, and therefore the wild-type form of LLT1 with His176 would have different crystal packing.

### 3.10. Comparison of LLT1, CD69 and Clr-g structures   

LLT1 shares relatively high three-dimensional structure similarity with other dimeric CTL proteins. All comparisons were performed with the LLT1_D2 coordinates. According to SSM structure superposition with *PDBeFold* (http://www.ebi.ac.uk/msd-srv/ssm/), LLT1 is structurally most similar to human CD69 (PDB entry 1e8i; Llera *et al.*, 2001 ▶; 36% sequence identity). Structure similarity to mouse Clr-g (PDB entry 3rs1; Skálová *et al.*, 2012 ▶; 44% sequence identity) has similar values when comparing monomers and is a little lower when comparing dimers. The parameters of the structure superpositions are as follows: LLT1 monomer/CD69 monomer, *Q*-score^1^ 0.82 and r.m.s. deviation 1.10 Å, aligned on 115 residues; LLT1 dimer/CD6 dimer, *Q*-score 0.67 and r.m.s. deviation 1.67 Å, aligned on 223 residues; LLT1 monomer/Clr-g monomer, *Q*-score 0.82 and r.m.s. deviation 0.96 Å, aligned on 116 residues; LLT1 dimer/Clr-g dimer, *Q*-score 0.55 and r.m.s. deviation 2.10 Å, aligned on 219 residues.

The comparison of the structure of LLT1_D2 with that of mouse Clr-g is shown in Fig. 2 ▶(*b*). The superposition was performed in *Coot* by SSM of chains *A*. There is an apparent difference in the mutual orientation of the monomers in the dimer of LLT1 in comparison with mouse Clr-g. There are shifts in the distant part of the long loop region of up to 5 Å. Differences are also present in the N- and C-terminal parts owing to the distinct lengths of the LLT1 and Clr-g constructs. Differences between LLT1 and CD69 are found in the same regions and are of the same character as the differences between LLT1 and Clr-g (not shown).

Electrostatic equipotential surfaces of LLT1 and mouse Clr-g (Skálová *et al.*, 2012 ▶) are shown in Fig. 2 ▶(*d*). There are some positive patches in the part distant from the N- and C-termini in LLT1. A similar and much stronger effect is observed for mouse Clr-g. The large patch in Clr-g is formed mainly owing to the contributions of Arg180, Arg193 and Arg198, which correspond to Glu162, Arg175 and Arg180 in LLT1. The change from Arg180 to Glu162 may explain the weaker positive patches in the case of LLT1.

### 3.11. Hypotheses about NKR-P1–LLT1 complexation   

The hexamer is formed by the packing of three ‘standard’ CTL dimers. To the best of our knowledge, no such molecular arrangement has been observed for CTL proteins. Each dimer in the hexamer interacts with two other dimers. The dimer–dimer interaction in the hexamer is analyzed in Figs. 6 ▶(*c*) and 6 ▶(*d*). Could this dimer–dimer interaction indicate the manner of NK CTL receptor–CTL ligand complex formation or other biologically relevant actions?

This interaction of LLT1–LLT1 dimers in the hexamer is not a ‘face-to-face’ interaction, as is classically expected for NK CTL receptor–CTL ligand interaction (Kamishikiryo *et al.*, 2011 ▶); rather, one monomer of the dimer binds to one dimer and the second monomer binds to another dimer of the hexamer. In this aspect it is similar to the model of the NKR-P1F–Clr-g complex (based on electrostatic complementarity) in our previous study (Skálová *et al.*, 2012 ▶).

The only experimentally determined structure of a CTL NK receptor–CTL ligand complex is the X-ray structure of the monomeric receptor NKp65–dimeric KACL ligand complex (PDB entry 4iop; Li *et al.*, 2013 ▶), where the binding mode is of the monomer–dimer type. The sequence identity of the CTL domain of LLT1 to the CTL domain of KACL is 49%, while that of LLT1 to NKp65 is 30%.

We have built several models of the NKR-P1–LLT1 complex. The modelling of NKR-P1 was performed in *SWISS-MODEL* (Biasini *et al.*, 2014 ▶) based on the crystal structure of mouse NKR-P1A (PDB entry 3m9z; Kolenko, Rozbeský, Vaněk, Kopecký *et al.*, 2011 ▶; 46% sequence identity). The crystal structure of mouse NKR-P1A (Kolenko, Rozbeský, Vaněk, Bezouška *et al.*, 2011 ▶; Kolenko, Rozbeský, Vaněk, Kopecký *et al.*, 2011 ▶) has extended flexible loops which form crystal contacts and most probably occupy a different position in solution (Sovová *et al.*, 2011 ▶). However, the identified receptor–ligand interaction pairs do not lie in the loops; therefore, it is possible to use the crystal structure as a modelling basis for our purposes.

The first model is based on the NKp65–KACL structure. Both the model of NKR-P1 and the structure of LLT1 were superimposed on the structure of the NKp65–KACL complex to simulate a model of the NKR-P1–LLT1 complex (Supplementary Fig. S1*a*). In this rather crude model it is possible to query the interaction between Lys169 of LLT1 and Glu205 of NKR-P1, which were identified by Kamishikiryo and coworkers as the key interacting residues based on SPR experiments (Kamishikiryo *et al.*, 2011 ▶). It should be noted that these SPR experiments were performed on nonglycosylated LLT1 produced in *E. coli*. In this first model, these two residues are 10 Å away from each other.

In the second model we exchanged the positions of the receptor and the ligand, *i.e.* NKR-P1 was superimposed on KACL and the individual chains of LLT1 on the positions of the monomers of NKp65 (Supplementary Fig. S1*b*). In this model, residues Lys169 of LLT1 and Glu205 of NKR-P1 are in direct contact.

The third model is based on the LLT1–LLT1 dimer–dimer mutual position in the hexameric structure. The NKR-P1 model was superimposed on the closest LLT1 chain in the hexamer with respect to a fixed LLT1 dimer (Supplementary Figs. S1*c* and S1*d*). In this model, the distance between LLT1 Lys169 and NKR-P1 Glu205 is 20 Å.

Based on the assumption of close positioning of Lys169 and Glu205, we conclude that the LLT1 arrangement in the hexameric packing is not a probable model for NK CTL receptor–CTL ligand interaction. Interestingly, the only model enabling direct contact of Lys169 and Glu205 residues is that which requires disruption of the LLT1 dimer into monomers. It remains to be shown experimentally in the future whether this is truly the case.

### 3.12. Oligomeric state of LLT1 in solution   

**Figure 7:**
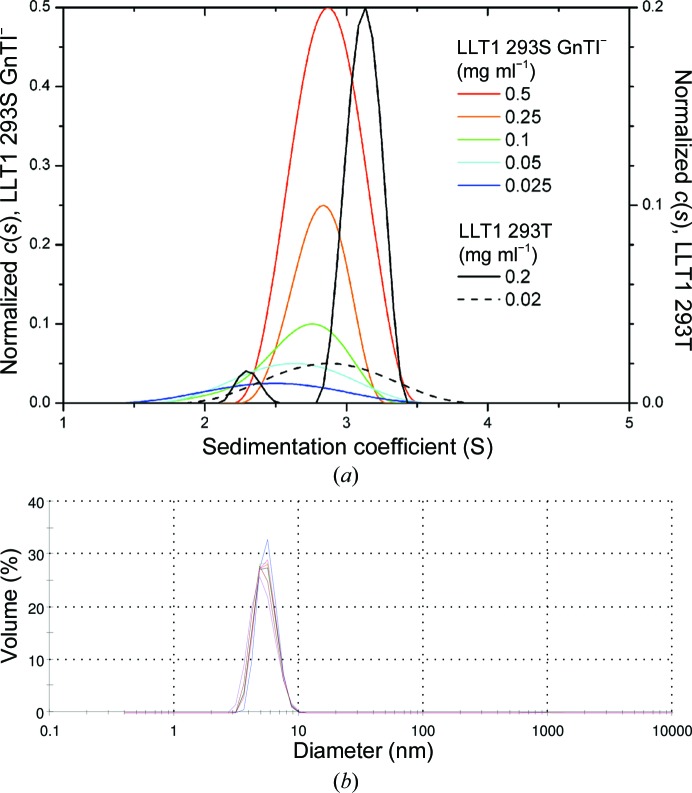
(*a*) Sedimentation analysis of LLT1 oligomerization. Glycosylated LLT1 with homogeneous GlcNAc_2_Man_5_ N-glycosylation produced in the HEK293S GnTI^−^ cell line was characterized by a sedimentation-velocity experiment in an analytical ultracentrifuge at five different concentrations (coloured lines). The continuous size distribution of the sedimenting species is shown, which was normalized with respect to the difference in loading concentration. A broad size distribution that is shifted towards lower values with lower protein concentration points to monomer–dimer equilibrium; for comparison, data for LLT1 produced in the HEK293T cell line with wild-type complex N-glycosylation are shown (black and dashed lines), with separated signals for dimeric and monomeric protein at higher protein concentration. (*b*) Size distribution of LLT1 measured by dynamic light scattering and scaled by volume. Seven measurements (distributions differentiated by colour) were performed with similar results, three of them at 291 K, two at 298 K and two at 303 K.

Sedimentation-equilibrium analysis has previously shown that LLT1 forms noncovalent dimers in solution that do not depend on its N-glycosylation (Bláha *et al.*, 2015 ▶). However, to assess for the presence of either monomers or higher oligomers (*e.g.* hexamers), we performed sedimentation-velocity measurements. Firstly, LLT1 produced in the HEK293T cell line with wild-type complex N-glycosylation was analyzed. At 0.2 mg ml^−1^ concentration, LLT1 behaves as two distinct species with *s*_20,w_ values of 2.43 and 3.14 ± 0.1 S corresponding to a monomer and a dimer, respectively (Fig. 7 ▶ *a*, black line). Upon tenfold dilution the two peaks merged into one with an average *s*_20,w_ value of 2.87 S, pointing to a monomer–dimer equilibrium at low concentration (Fig. 7 ▶ *a*, dashed line). No higher oligomers were detected. LLT1 with homogeneous GlcNAc_2_Man_5_ N-glycosylation produced in the HEK293S GnTI^−^ cell line behaved similarly, yielding broad size distributions that shifted towards lower sedimentation-coefficient values at lower protein concentrations (Fig. 7 ▶ *a*, coloured lines), thus reflecting monomer–dimer equilibrium behaviour of the protein. The fact that monomeric and dimeric forms are not separated in these distributions might suggest that LLT1 produced with shorter *N*-glycans dimerizes more weakly and/or that equilibrium exchange is faster than for LLT1 with wild-type complex *N*-glycans, with an estimated *K*_d_ lying in the low micromolar range (∼20 µ*M*).

Dynamic light scattering was performed for LLT1 with homogeneous GlcNAc_2_Man_5_ N-glycosylation produced in the HEK293S GnTI^−^ cell line at 1 mg ml^−1^ concentration (Fig. 7 ▶ *b*). The experiment was repeated at temperatures of 291, 298 and 303 K. Particles with a diameter of 55 ± 10 Å were observed in all cases. This value corresponds to a Stokes diameter of 56 Å for the LLT1 dimer, as computed based on the three-dimensional structure of LLT1 in *HYDROPRO* (Ortega *et al.*, 2011 ▶), while the Stokes diameter computed for the LLT1 hexamer is 70 Å and that for the monomer is 20 Å.

## 4. Conclusions   

This study has introduced the first X-ray structures of LLT1. A new conformation of the outer loop in the long loop region of a CTL receptor/ligand was observed; its newly observed position near α-helix 96–104 is probably the result of conformational sampling with satisfied packing positions of the neighbours and is without any special biological significance. Furthermore, the four structures with their mutual differences extend our understanding of the influence of glycosylation on the structure and the role of flexibility of the dimer interface in complex formation.

Glycosylation control has been shown by this study to play a very important role in structural investigations of small mammalian proteins. The fully homogeneously glycosylated extracellular domain of human LLT1 clearly gained from the uniformity of the glycosylation pattern. The longer, yet homogeneous, oligosaccharides seem to participate in crystal formation of the LLT1_glyco form. Their uniform length and type are important. It is also clear that these longer oligosaccharides would disable the crystal contacts observed for deglycosylated LLT1 in the dimeric structures LLT1_D1 and LLT1_D2. The deglycosylated protein variant enables the formation of contacts in which GlcNAc plays an important role, but longer antennae would disable such tight packing into a crystal. Both forms (uniformly glycosylated and deglycosylated) proved to be important for the structural results, thereby showing the extreme importance of controlled post-translational modifications for detailed molecular studies.

The hydrogen-bonding pattern at the LLT1 dimer interface is not the governing interaction in dimerization and can be significantly weakened, as is the case for the LLT1_glyco form. Here, the interactions with the neighbouring dimers in the hexamer are strong enough to slightly deform the dimer so that several interface hydrogen bonds near the termini region disappear (the hydrophobic core remains unchanged). The variability of the CTL receptor or ligand dimer interface reported previously, and confirmed by this study, is thus clearly related to the CTL dimer–dimer interaction for the first time. The capacity of these small proteins to form very flexible but still well defined dimers is very likely to form a part of their overall strategy for mediating plastic cell–cell interactions in the immune system.

## Supplementary Material

PDB reference: human LLT1, dimeric form, 4qkh

PDB reference: 4qki

PDB reference: hexameric form, 4qkj

PDB reference: monomeric form, 4qkg

Supporting Information.. DOI: 10.1107/S1399004714027928/rr5085sup1.pdf

## Figures and Tables

**Table 1:** Data-collection statistics and structure-refinement parameters Values in parentheses are for the highest resolution shell.

	LLT1_mono	LLT1_D1	LLT1_D2	LLT1_glyco
PDB code	4qkg	4qkh	4qki	4qkj
pH of crystallization condition	3.5	7.0	7.0	4.2
Glycosylation	GlcNAc	GlcNAc	GlcNAc	GlcNAc_2_Man_5_
Oligomer	Monomer	Dimer	Dimer	Hexamer
Data-collection statistics				
Resolution range ()	50.01.95 (1.981.95)	47.31.8 (1.841.80)	43.71.8 (1.841.80)	39.02.7 (2.762.70)
Space group	*P*3_2_21	*P*2_1_2_1_2_1_	*P*2_1_2_1_2_1_	*P*6_3_22
Unit-cell parameters ()	*a* = *b* = 47.3, *c* = 106.1	*a* = 50.9, *b* = 57.8, *c* = 82.3	*a* = 51.3, *b* = 54.1, *c* = 74.2	*a* = *b* = 70.1, *c* = 101.7
Radiation source	I02, DLS	I04-1, DLS	I04-1, DLS	BM 14.1, BESSY II
Detector	ADSC Q315 CCD	PILATUS 2M	PILATUS 2M	MAR Mosaic 225 CCD
Data-processing software	*HKL*-2000 [*DENZO*, *SCALEPACK*]	*MOSFLM*, *AIMLESS*	*MOSFLM*, *AIMLESS*	*HKL*-2000 [*DENZO*, *SCALEPACK*], anisotropy server
Wavelength ()	0.9796	0.9200	0.9200	0.9184
No. of images	220	900	1800	80 [images 150, 330360]
Crystal-to-detector distance (mm)	256.1	325.2	251.9	288.1
Exposure time per image (s)	1	0.2	0.2	2
Oscillation width ()	0.5	0.2	0.2	0.5
No. of observations	66670 (3340)	175131 (5869)	113353 (6521)	478731 (1201)
No. of unique reflections	10555 (506)	21104 (1087)	18996 (1050)	4431 (273)
Data completeness (%)	99.7 (100)	91.8 (81.0)	96.7 (91.6)	99.5 (99.3)
Average multiplicity	6.3 (6.6)	8.3 (5.4)	6.0 (6.2)	4.3 (4.4)
Mosaicity ()	0.30.7	1.8	1.3	0.81.4
*I*/(*I*)	21.2 (2.9)	11.3 (3.0)	11.8 (3.7)	16.2 (2.0)
Solvent content (%)	44	36	25	46
Matthews coefficient (^3^Da^1^)	2.18	1.93	1.64	2.30
*B* factor from Wilson plot (^2^)	34.0	20.7	17.7	66.1
*R*_merge_ †	0.074 (0.599)	0.093 (0.462)	0.082 (0.404)	0.096 (0.757)
*R*_p.i.m._ ‡	0.032 (0.251)	0.045 (0.309)	0.053 (0.257)	0.031 (0.171)
Structure parameters				
*R*_work_ § (%)	19.4	17.5	17.8	22.1
*R*_free_ § (%)	24.7	25.0	24.4	29.8
*R*_all_ § (%)	20.1	18.0	18.3	23.1
Average *B* factor (^2^)	39.8	27.6	20.9	40.1
R.m.s.d. from ideal bond lengths ()	0.016	0.016	0.016	0.017
R.m.s.d. from ideal bond angles ()	1.9	1.7	1.8	2.0
No. of monomers per asymmetric unit	1	2	2	1
Amino-acid residues located	*A*72*A*146, *A*161*A*193	*A*70*A*192, *B*72*B*192	*A*73*A*194, *B*73*B*192	*A*74*A*188
Asn residues with located GlcNAc	*A*95	*A*95, *A*147, *B*95, *B*147	*A*95, *A*147, *B*95, *B*147	*A*95
No. of water molecules	42	215	167	15
Other localized moieties	1 SO_4_^2^			
Ramachandran statistics: residues in favoured region (%)	95	98	98	87

† *R*_merge_ = 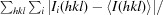 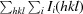 (Diederichs Karplus, 1997 ▶), where *I_i_*(*hkl*) and *I*(*hkl*) are the observed individual and mean intensities of a reflection with indices *hkl*, respectively, 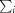 is the sum over the individual measurements of a reflection with indices *hkl* and 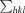 is the sum over all reflections.

‡ *R*_p.i.m._ = 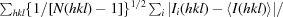 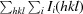 (Weiss, 2001 ▶), where *N*(*hkl*) is the redundancy of the reflection with indices *hkl*.

§ *R*_work_ = 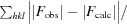 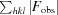, where *F*_obs_ and *F*_calc_ are the observed and calculated structure-factor amplitudes for the reflection with indices *hkl* for the working set of reflections. *R*_free_ is the same as *R*_work_ but is calculated for 56% of the data omitted from refinement. *R*_all_ sums over all reflections.

## Footnotes

1
*Q*-score is the default sorting criterion of structure similarity in *PDBeFold* and is defined as *Q* = *N*_algn_ × *N*_algn_/{[1 + (r.m.s.d./*R*_0_)^2^] × *N*_res1_ × *N*_res2_}, where *R*_0_ = 3 Å, *N*_res1_ and *N*_res2_ are the numbers of residues in the overlapping structures and *N*_algn_ is the number of overlapped residues.
