# Supplementary material for: Four crystal structures of human LLT1, a ligand of human NKR-P1, in varied glycosylation and oligomerization states
Source: Acta Crystallogr D Biol Crystallogr. 2015 Feb 26;71(Pt 3):578–91. doi: 10.1107/S1399004714027928 (PMC4356368; doi:10.1107/S1399004714027928)
Supplement: Supplementary file 1 [file d-71-00578-sup1.pdf]

BIOLOGICAL CRYSTALLOGRAPHY

Volume 71 (2015)

Supporting information for article:

Four crystal structures of human LLT1, a ligand for human NKR-P1, in varied glycosylation and oligomerization states

Tereza Skálová, Jan Bláha, Karl Harlos, Jarmila Dušková, Tomáš Koval', Jan Stránský, Jindřich Hašek, Ondřej Vaněk and Jan Dohnálek

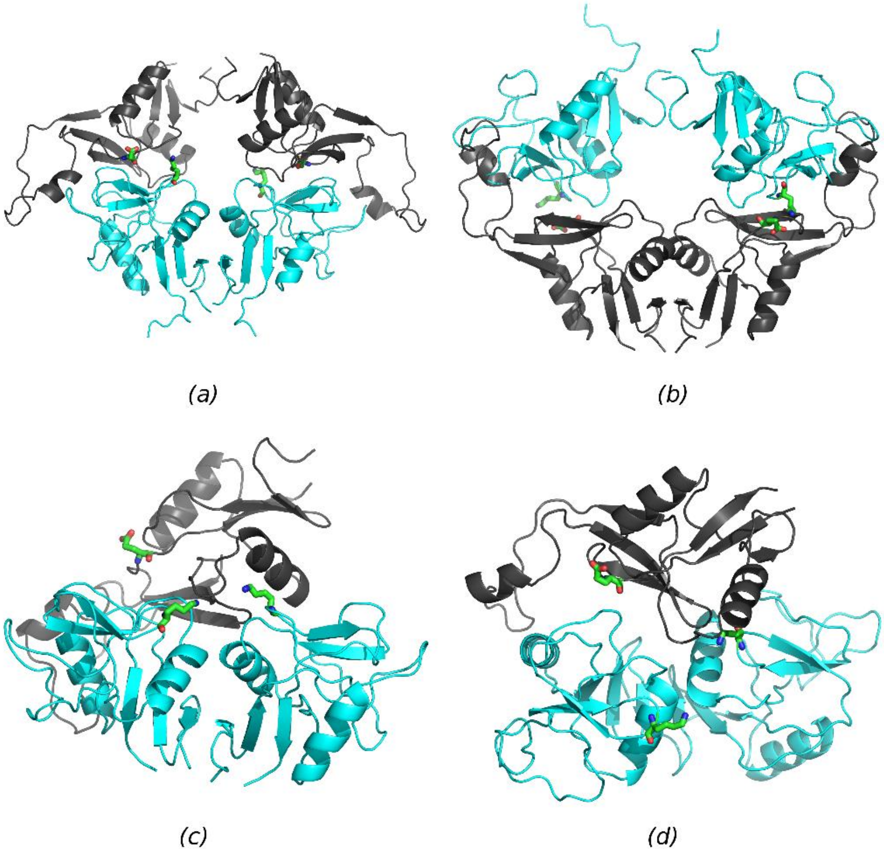

Figure S1 Discussed models of NKR-P1:LLT1 interaction. LLT1 cyan, model of NKR-P1 black. Residues LLT1 Lys169 and NKR-P1 Glu205 important for the interaction (Kamishikiryo *et al.*, 2011) are shown as green sticks. (*a*) Model based on the NKp65:KACL structure (PDB code 4iop, Li *et al.*, 2013), NKR-P1 is placed in position of NKp65 and LLT1 in position of KACL. (*b*) Model based on the same structure, but NKR-P1 takes the position of KACL and LLT1 of NKp65. (*c*, *d*) Model based on hexameric packing of LLT1_glyco. LLT1 dimer is in its position from the observed hexamer and NKR-P1 in the position of the closest LLT1 monomer in the hexamer. (*d*) A view from the “top” – from the termini distal side.
